# Supplementary material for: Emergence and Spread of the SARS-CoV-2 Variant of Concern Delta across Different Brazilian Regions
Source: Microbiol Spectr. 2022 Aug 24;10(5):e02641-21. doi: 10.1128/spectrum.02641-21 (PMC9604183; doi:10.1128/spectrum.02641-21)
Supplement: Supplemental file 1 — Supplemental material. Download spectrum.02641-21-s0001.pdf, PDF file, 0.5 MB [file spectrum.02641-21-s0001.pdf]

SUPPLEMENTARY TABLES

SUPPLEMENTARY TABLE 1: Distribution SARS-CoV-2 VOC Delta Dataset from Brazil's Five regions Across the Country's Three Main Clusters

Cluster	BR (N/N%)		N (N/N%)		NE (N/N%)		CW (N/N%)		SE (N/N%)		S (N/N%)	
**Main**	343	15,15	1	4.35	17	24.29	18	33.96	283	15.44	24	10.21
**BR-I**	1545	68,24	21	91.30	50	71.43	32	60.38	1,396	73.43	46	19.57
**AY.101**	205	9,05	0	0.00	2	2.86	1	1.89	41	2.24	161	68.51
**AY.46.3**	171	7,55	1	4.35	1	1.43	2	3.77	163	8.89	4	1.70
**Total**	2264	100	23	100	70	100	53	100	1,883	100	235	100

The table details the distribution of sampled genomes from Brazil's Five regions across its SARS-CoV-2 VOC Delta main three and major clusters, BR-I (*n* = 1,560), AY.101 (*n* = 207), and AY.46.3 (*n* = 171). BR: Brazil, CW: Central-western, N: North, NE: Northeast, SE: Southeast, S: South.

SUPPLEMENTARY TABLE 2: SARS-CoV-2 VOC Delta Clusterization Profile

Clusterization	BR (N/N%)		N (N/N%)		NE (N/N%)		CW (N/N%)		SE (N/N%)		S (N/N%)	
**Unclustered**	207	9.14	16	69.57	45	64.29	16	30.19	96	5.10	34	14.47
**Clustered**	2057	90.86	7	30.43	25	35.71	37	69.81	1787	94.90	201	85.53
**Total**	2264	100	23	100	70	100	53	100	1883	100	235	100

The table details the clusterization profile of genomes sampled in Brazil (BR, *n* = 2,264) and its North (N, *n* = 23), Northeast (NE, *n* = 70), Central-western (CW, *n* = 53), Southeast (SE, *n* = 1,883), and South (S, *n* = 235) regions. Phylogenetic clusters were defined as any statistically supported (aLRT ≥ 75) group of more than one sequence from the same geographical region. BR: Brazil, CW: Central-western, N: North, NE: Northeast, SE: Southeast, S: South.

SUPPLEMENTARY FIGURES

SUPPLEMENTARY FIGURE 1

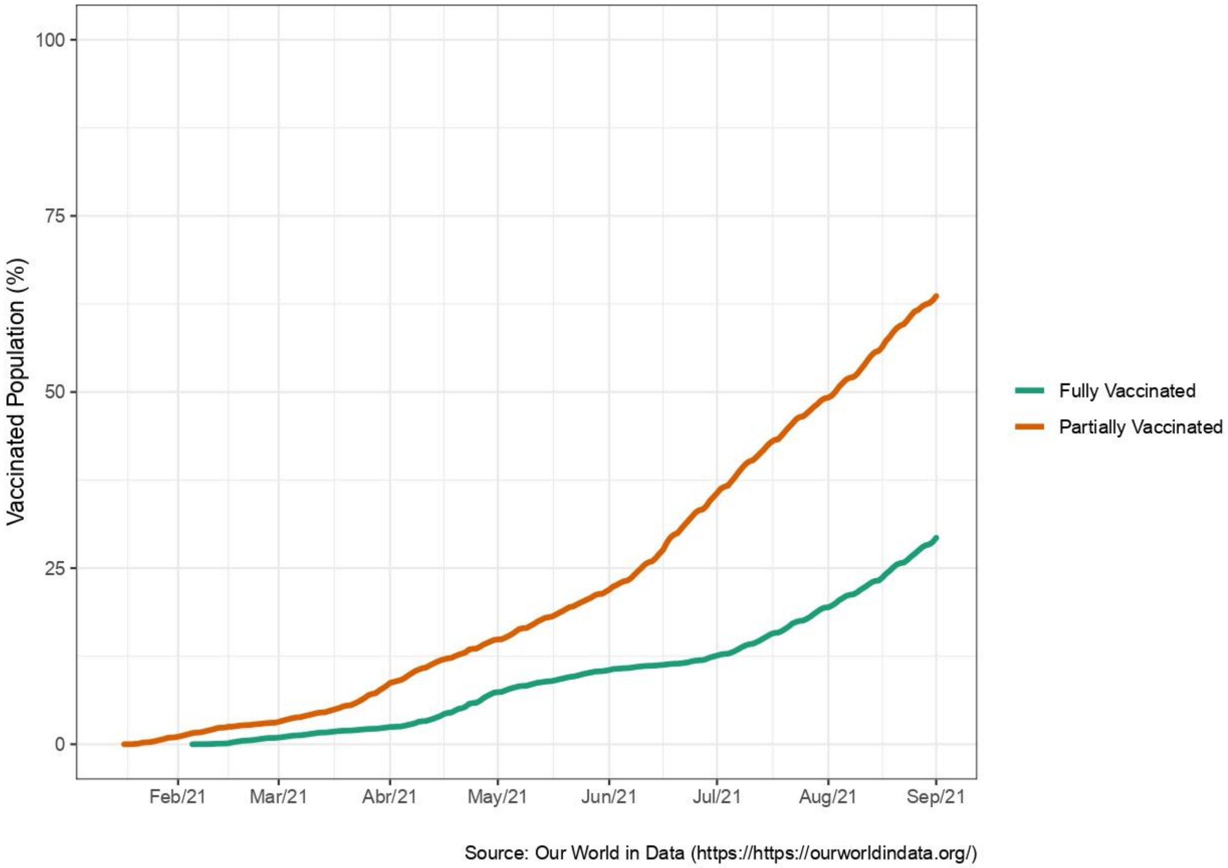

Vaccination status evolution in the brazzilian population. The graph depicts the the share of brazilian population single and fully vaccinated from February/2021 to September 2021.

APPENDIX 1

COVID-19 Fiocruz Genomic Surveillance Network

Carlos Leonardo Araújo<br>Cleber Furtado Akesenen<br>Fernando Braga Stehling Dias<br>Igor Oliveira Duarte<br>Jamille Mendes Bezerra<br>Joaquim Cesar Sousa Jr<br>Pedro Miguel Carneiro Jerônimo<br>Suzana Almeida Porto<br>Thaís de Oliveira Costa<br>Thais Ferreira de Oliveira<br>Ticiane Cavalcante de Souza<br>Veridiana Pessoa Miyajima	*Analytical Competence Molecular Epidemiology Laboratory (ACME), Fundação Oswaldo Cruz (FIOCRUZ) – Ceará, Brasil*
Acacia Lourenço Francisco Nasr<br>Ana Carolina De la Vechia<br>Rosana Aparecida Piler<br>Tatiane Motta Huggler	*Divisão de Vigilância de Doenças Transmissíveis, Secretaria Estadual de Saúde do Paraná – Paraná, Brasil*
Cristiano Fernandes	*Fundação de Vigilância em Saúde do Amazonas - Dra. Rosemary Costa Pinto – Amazonas, Brasil*
Marcelo Gomes	*Grupo de Métodos Analíticos em Vigilância Epidemiológica, Programa de Computação Científica (PROCC), Fundação Oswaldo Cruz (FIOCRUZ) – Rio de Janeiro, Brasil*
Adriano Abbud<br>Katia Oliveira Correa	*Instituto Adolfo Lutz – São Paulo, Brasil*
Alexandre Freitas da Silva<br>Antonio Marinho da Silva Neto<br>Cássia Docena<br>Filipe Zimmer Dezordi<br>Gustavo Barbosa de Lima<br>Laís Ceschini Machado<br>Lilian Caroliny Amorim Silva<br>Marcelo Henrique Santos Paiva<br>Matheus Filgueira Bezerra<br>Raul Emídio de Lima	*Instituto Aggeu Magalhães, Fundação Oswaldo Cruz (FIOCRUZ) – Pernambuco, Brasil*
Andreia Akemi Suzukawa<br>Mauro de Medeiros Oliveira<br>Michelle Orane Schemberger	*Instituto Carlos Chagas (ICC), Fundação Oswaldo Cruz (FIOCRUZ) – Paraná, Brasil*
Beatriz Grinsztejn<br>Patricia Brasil<br>Valdiléa G Veloso	*Instituto Nacional de Infectologia Evandro Chagas (INI), Fundação Oswaldo Cruz (FIOCRUZ) – Rio de Janeiro, Brasil*
Felicidade Pereira	*Laboratorio Central de Saúde Pública da Bahia (LACEN-BA) – Bahia, Brasil*
Dalane Loudal Florentino Teixeira<br>Haline Barroso	*Laboratório Central de Saúde Pública da Paraíba (LACEN-PB) – Paraíba, Brasil*
Anderson Brandao Leite	*Laboratório Central de Saúde Pública de Alagoas (LACEN-AL) – Alagoas, Brasil*
Vinicius Lemes da Silva	*Laboratorio Central de Saúde Pública de Goiás (LACEN-GO) – Goiás, Brasil*
André Felipe Leal Bernardes<br>Felipe Campos de Melo Iani	*Laboratório Central de Saúde Pública de Minas Gerais (LACEN-MG) – Minas Gerais, Brasil*
Irina Riediger<br>Maria do Carmo Debur	*Laboratorio Central de Saúde Pública de Paraná (LACEN-PR) – Paraná, Brasil*
	Laboratorio Central de Saúde Pública de Pernambuco (LACEN-PE) – Pernambuco, Brasil
Themis Rocha	*Laboratorio Central de Saúde Pública de Sergipe (LACEN-RN) – Rio Grande do Norte, Brasil*
Andreia Santos Costa<br>Lindomar dos Anjos Silva	*Laboratório Central de Saúde Pública do Amapá (LACEN-AP) – Amapá, Brasil*
Tirza Peixoto Mattos	*Laboratório Central de Saúde Pública do Amazonas (LACEN-AM) – Amazonas, Brasil*
Ana Barjud Marques Maximo<br>Liana Perdigão Mello<br>Vania Angelica Feitosa Viana	*Laboratório Central de Saúde Pública do Ceará (LACEN-CE) – Ceará, Brasil*

Rodrigo Ribeiro Rodrigues	*Laboratório Central de Saúde Pública do Espirito Santo (LACEN-ES) – Espírito Santo, Brasil*
Darcita Buerger Rovaris<br>Sandra Bianchini Fernandes	*Laboratorio Central de Saúde Pública do Estado de Santa Catarina (LACEN-SC) – Santa Catarina, Brasil*
Lidio Gonçalves Lima Neto	*Laboratório Central de Saúde Pública do Maranhão (LACEN-MA) – Maranhão, Brasil*
Valnete Andrade	*Laboratório Central de Saúde Pública do Pará (LACEN-PA) – Pará, Brasil*
Andrea Cony Cavalcanti	*Laboratório Central de Saúde Pública do Rio de Janeiro (LACEN-RJ) – Rio de Janeiro, Brasil*
Richard Steiner Salvato<br>Tatiana Schäffer Gregianini	*Laboratório Central de Saúde Pública do Rio Grande do Sul (LACEN-RS) – Rio Grande do Sul, Brasil*
Jucimária Dantas Galvão	*Laboratorio Central de Saúde Pública do Tocantins (LACEN-TO) – Tocantins, Brasil*
Ágatha Costa<br>André de Lima Guerra Corado<br>Fernanda Nascimento<br>George Silva<br>Karina Pessoa<br>Luciana Fé Gonçalves<br>Maria Júlia Brandão<br>Matilde Mejía<br>Michele Silva de Jesus<br>Valdinete Alves Nascimento<br>Victor Souza	*Laboratório de Ecologia de Doenças Transmissíveis na Amazônia (EDTA), Instituto Leônidas e Maria Deane, Fundação Oswaldo Cruz (FIOCRUZ) – Amazonas, Brasil*
Bruna Mendonça da Silva<br>Fernando do Couto Motta<br>Jéssica de Macedo Carvalho<br>Larissa Macedo Pinto	*Laboratório de Vírus Respiratórios e Sarampo, Instituto Oswaldo Cruz (IOC), Fundação Oswaldo Cruz (FIOCRUZ) – Rio de Janeiro, Brasil*
Fernando Vinhal	*Laboratório HLAGYN – Goiás, Brasil*
Isabela de Lucena Heráclio<br>Morgana de Freitas Caraciolo<br>Roberta Mendes Abreu Silva<br>Silvio Rodrigues de Almeida<br>Thayna Karoline Sousa Silva	*Programa de Treinamento em Epidemiologia Aplicada (EpiSUS-Avançado), Ministério da Saúde – Brasil*
Alessandro Álvares Magalhães<br>Érika Lopes Rocha Batista	*Secretaria de Saúde de Aparecida de Goiânia – Goiás, Brasil*
Greice Madeleine Ikeda do Carmo<br>Janaína Sallas<br>Walquiria Aparecida Almeida	*Secretaria de Vigilância em Saúde, Ministério da Saúde – Brasil*
Marcio Garcia<br>Cecilia Leite Costa<br>Eduardo Ruback dos Santos	*Secretaria de Vigilância em Saúde, Secretaria Municipal de Saúde – Rio de Janeiro, Brasil*<br>*Unidade de Apoio Diagnostico (UNADIG), Fundação Oswaldo Cruz (FIOCRUZ) – Ceará, Brasil*
João Felipe Bezerra	*Universidade Federal da Paraíba (UFPB) – Paraíba, Brasil*

APPENDIX 2

Sequences Produced by the COVID-19 Fiocruz Genomic Surveillance Network

EPI_ISL_2311861	EPI_ISL_3536535	EPI_ISL_3540020	EPI_ISL_3801803	EPI_ISL_3801843	EPI_ISL_3801883
EPI_ISL_2443545	EPI_ISL_3539829	EPI_ISL_3540021	EPI_ISL_3801804	EPI_ISL_3801844	EPI_ISL_3801884
EPI_ISL_2466268	EPI_ISL_3539830	EPI_ISL_3540022	EPI_ISL_3801805	EPI_ISL_3801845	EPI_ISL_3801885
EPI_ISL_2645411	EPI_ISL_3539906	EPI_ISL_3540023	EPI_ISL_3801806	EPI_ISL_3801846	EPI_ISL_3801886
EPI_ISL_2645412	EPI_ISL_3539907	EPI_ISL_3540024	EPI_ISL_3801807	EPI_ISL_3801847	EPI_ISL_3801887
EPI_ISL_2645413	EPI_ISL_3539908	EPI_ISL_3540025	EPI_ISL_3801808	EPI_ISL_3801848	EPI_ISL_3801888
EPI_ISL_2645414	EPI_ISL_3539909	EPI_ISL_3540026	EPI_ISL_3801809	EPI_ISL_3801849	EPI_ISL_3801889
EPI_ISL_2645415	EPI_ISL_3539910	EPI_ISL_3540027	EPI_ISL_3801810	EPI_ISL_3801850	EPI_ISL_3801890
EPI_ISL_2677318	EPI_ISL_3539911	EPI_ISL_3540028	EPI_ISL_3801811	EPI_ISL_3801851	EPI_ISL_3801891
EPI_ISL_2731451	EPI_ISL_3539912	EPI_ISL_3540029	EPI_ISL_3801812	EPI_ISL_3801852	EPI_ISL_3801892
EPI_ISL_2731452	EPI_ISL_3539913	EPI_ISL_3540030	EPI_ISL_3801813	EPI_ISL_3801853	EPI_ISL_3801893
EPI_ISL_2863898	EPI_ISL_3539914	EPI_ISL_3540031	EPI_ISL_3801814	EPI_ISL_3801854	EPI_ISL_3801894
EPI_ISL_2863899	EPI_ISL_3539915	EPI_ISL_3540032	EPI_ISL_3801815	EPI_ISL_3801855	EPI_ISL_3801895
EPI_ISL_2982735	EPI_ISL_3539916	EPI_ISL_3540033	EPI_ISL_3801816	EPI_ISL_3801856	EPI_ISL_3801896
EPI_ISL_2982743	EPI_ISL_3539917	EPI_ISL_3540034	EPI_ISL_3801817	EPI_ISL_3801857	EPI_ISL_3801897
EPI_ISL_2983373	EPI_ISL_3539918	EPI_ISL_3540035	EPI_ISL_3801818	EPI_ISL_3801858	EPI_ISL_3801898
EPI_ISL_2983452	EPI_ISL_3539919	EPI_ISL_3540036	EPI_ISL_3801819	EPI_ISL_3801859	EPI_ISL_3801899
EPI_ISL_3134853	EPI_ISL_3539920	EPI_ISL_3540037	EPI_ISL_3801820	EPI_ISL_3801860	EPI_ISL_3801900
EPI_ISL_3134854	EPI_ISL_3539921	EPI_ISL_3540038	EPI_ISL_3801821	EPI_ISL_3801861	EPI_ISL_3801901
EPI_ISL_3134855	EPI_ISL_3539923	EPI_ISL_3540039	EPI_ISL_3801822	EPI_ISL_3801862	EPI_ISL_3801902
EPI_ISL_3274747	EPI_ISL_3539924	EPI_ISL_3703695	EPI_ISL_3801823	EPI_ISL_3801863	EPI_ISL_3801903
EPI_ISL_3274750	EPI_ISL_3540001	EPI_ISL_3703704	EPI_ISL_3801824	EPI_ISL_3801864	EPI_ISL_3801904
EPI_ISL_3274764	EPI_ISL_3540002	EPI_ISL_3758076	EPI_ISL_3801825	EPI_ISL_3801865	EPI_ISL_3801905
EPI_ISL_3274790	EPI_ISL_3540003	EPI_ISL_3758085	EPI_ISL_3801826	EPI_ISL_3801866	EPI_ISL_3801906
EPI_ISL_3386143	EPI_ISL_3540004	EPI_ISL_3758115	EPI_ISL_3801827	EPI_ISL_3801867	EPI_ISL_3801907
EPI_ISL_3447445	EPI_ISL_3540005	EPI_ISL_3758116	EPI_ISL_3801828	EPI_ISL_3801868	EPI_ISL_3801908
EPI_ISL_3447452	EPI_ISL_3540006	EPI_ISL_3758124	EPI_ISL_3801829	EPI_ISL_3801869	EPI_ISL_3801909
EPI_ISL_3536204	EPI_ISL_3540007	EPI_ISL_3758134	EPI_ISL_3801830	EPI_ISL_3801870	EPI_ISL_3801910
EPI_ISL_3536331	EPI_ISL_3540008	EPI_ISL_3758136	EPI_ISL_3801831	EPI_ISL_3801871	EPI_ISL_3801911
EPI_ISL_3536483	EPI_ISL_3540009	EPI_ISL_3758138	EPI_ISL_3801832	EPI_ISL_3801872	EPI_ISL_3801912
EPI_ISL_3536492	EPI_ISL_3540010	EPI_ISL_3758144	EPI_ISL_3801833	EPI_ISL_3801873	EPI_ISL_3801913
EPI_ISL_3536501	EPI_ISL_3540011	EPI_ISL_3758159	EPI_ISL_3801834	EPI_ISL_3801874	EPI_ISL_3801914
EPI_ISL_3536502	EPI_ISL_3540012	EPI_ISL_3801795	EPI_ISL_3801835	EPI_ISL_3801875	EPI_ISL_3801915
EPI_ISL_3536503	EPI_ISL_3540013	EPI_ISL_3801796	EPI_ISL_3801836	EPI_ISL_3801876	EPI_ISL_3801916
EPI_ISL_3536504	EPI_ISL_3540014	EPI_ISL_3801797	EPI_ISL_3801837	EPI_ISL_3801877	EPI_ISL_3801917
EPI_ISL_3536512	EPI_ISL_3540015	EPI_ISL_3801798	EPI_ISL_3801838	EPI_ISL_3801878	EPI_ISL_3801918
EPI_ISL_3536519	EPI_ISL_3540016	EPI_ISL_3801799	EPI_ISL_3801839	EPI_ISL_3801879	EPI_ISL_3801919
EPI_ISL_3536522	EPI_ISL_3540017	EPI_ISL_3801800	EPI_ISL_3801840	EPI_ISL_3801880	EPI_ISL_3801920
EPI_ISL_3536527	EPI_ISL_3540018	EPI_ISL_3801801	EPI_ISL_3801841	EPI_ISL_3801881	EPI_ISL_3801921
EPI_ISL_3536531	EPI_ISL_3540019	EPI_ISL_3801802	EPI_ISL_3801842	EPI_ISL_3801882	EPI_ISL_3801922
EPI_ISL_3801923	EPI_ISL_3803003	EPI_ISL_3832332	EPI_ISL_3832374	EPI_ISL_3835281	EPI_ISL_3922261
EPI_ISL_3801924	EPI_ISL_3825472	EPI_ISL_3832333	EPI_ISL_3832375	EPI_ISL_3835287	EPI_ISL_3944568

EPI_ISL_3801925	EPI_ISL_3827877	EPI_ISL_3832334	EPI_ISL_3832376	EPI_ISL_3835358	EPI_ISL_3944569
EPI_ISL_3801926	EPI_ISL_3827878	EPI_ISL_3832335	EPI_ISL_3832377	EPI_ISL_3841246	EPI_ISL_3944570
EPI_ISL_3801927	EPI_ISL_3827879	EPI_ISL_3832336	EPI_ISL_3832378	EPI_ISL_3841247	EPI_ISL_4080931
EPI_ISL_3801928	EPI_ISL_3827892	EPI_ISL_3832337	EPI_ISL_3832379	EPI_ISL_3841248	EPI_ISL_4080932
EPI_ISL_3801929	EPI_ISL_3827902	EPI_ISL_3832338	EPI_ISL_3832380	EPI_ISL_3841249	EPI_ISL_4080933
EPI_ISL_3801930	EPI_ISL_3827916	EPI_ISL_3832339	EPI_ISL_3832381	EPI_ISL_3841250	EPI_ISL_4515966
EPI_ISL_3801931	EPI_ISL_3827920	EPI_ISL_3832340	EPI_ISL_3832382	EPI_ISL_3912055	EPI_ISL_4520335
EPI_ISL_3801932	EPI_ISL_3827922	EPI_ISL_3832341	EPI_ISL_3832383	EPI_ISL_3912077	EPI_ISL_4520342
EPI_ISL_3801933	EPI_ISL_3827924	EPI_ISL_3832342	EPI_ISL_3832384	EPI_ISL_3912386	EPI_ISL_5490956
EPI_ISL_3801934	EPI_ISL_3827926	EPI_ISL_3832343	EPI_ISL_3832385	EPI_ISL_3912389	EPI_ISL_5490974
EPI_ISL_3801935	EPI_ISL_3827929	EPI_ISL_3832344	EPI_ISL_3832386	EPI_ISL_3912391	EPI_ISL_5491028
EPI_ISL_3801936	EPI_ISL_3827930	EPI_ISL_3832345	EPI_ISL_3832387	EPI_ISL_3912392	EPI_ISL_5491035
EPI_ISL_3801937	EPI_ISL_3827933	EPI_ISL_3832346	EPI_ISL_3832388	EPI_ISL_3912393	EPI_ISL_5491128
EPI_ISL_3801938	EPI_ISL_3827935	EPI_ISL_3832347	EPI_ISL_3832389	EPI_ISL_3912394	EPI_ISL_5491139
EPI_ISL_3802976	EPI_ISL_3827936	EPI_ISL_3832348	EPI_ISL_3832390	EPI_ISL_3912395	EPI_ISL_6100863
EPI_ISL_3802977	EPI_ISL_3827939	EPI_ISL_3832349	EPI_ISL_3832391	EPI_ISL_3912396	EPI_ISL_6100864
EPI_ISL_3802978	EPI_ISL_3827940	EPI_ISL_3832350	EPI_ISL_3832392	EPI_ISL_3912397	EPI_ISL_6100865
EPI_ISL_3802979	EPI_ISL_3827943	EPI_ISL_3832351	EPI_ISL_3832393	EPI_ISL_3912398	EPI_ISL_6100866
EPI_ISL_3802980	EPI_ISL_3827944	EPI_ISL_3832352	EPI_ISL_3832394	EPI_ISL_3912399	EPI_ISL_6100867
EPI_ISL_3802981	EPI_ISL_3827945	EPI_ISL_3832353	EPI_ISL_3832447	EPI_ISL_3912400	EPI_ISL_6100868
EPI_ISL_3802982	EPI_ISL_3827946	EPI_ISL_3832354	EPI_ISL_3832448	EPI_ISL_3912401	EPI_ISL_6100869
EPI_ISL_3802983	EPI_ISL_3827948	EPI_ISL_3832355	EPI_ISL_3832449	EPI_ISL_3912402	EPI_ISL_6100870
EPI_ISL_3802984	EPI_ISL_3827949	EPI_ISL_3832356	EPI_ISL_3832450	EPI_ISL_3912403	EPI_ISL_6100871
EPI_ISL_3802985	EPI_ISL_3827950	EPI_ISL_3832357	EPI_ISL_3832451	EPI_ISL_3912404	EPI_ISL_6100876
EPI_ISL_3802986	EPI_ISL_3827951	EPI_ISL_3832358	EPI_ISL_3832452	EPI_ISL_3912406	EPI_ISL_6100877
EPI_ISL_3802987	EPI_ISL_3827953	EPI_ISL_3832359	EPI_ISL_3832453	EPI_ISL_3914048	EPI_ISL_6100878
EPI_ISL_3802988	EPI_ISL_3827954	EPI_ISL_3832360	EPI_ISL_3832454	EPI_ISL_3914214	EPI_ISL_6100879
EPI_ISL_3802989	EPI_ISL_3827955	EPI_ISL_3832361	EPI_ISL_3832460	EPI_ISL_3914313	EPI_ISL_6100880
EPI_ISL_3802990	EPI_ISL_3827957	EPI_ISL_3832362	EPI_ISL_3832462	EPI_ISL_3922205	EPI_ISL_6100881
EPI_ISL_3802991	EPI_ISL_3827958	EPI_ISL_3832363	EPI_ISL_3832464	EPI_ISL_3922214	EPI_ISL_6100882
EPI_ISL_3802992	EPI_ISL_3827963	EPI_ISL_3832364	EPI_ISL_3832465	EPI_ISL_3922218	
EPI_ISL_3802993	EPI_ISL_3827969	EPI_ISL_3832365	EPI_ISL_3832466	EPI_ISL_3922221	
EPI_ISL_3802994	EPI_ISL_3827970	EPI_ISL_3832366	EPI_ISL_3832469	EPI_ISL_3922225	
EPI_ISL_3802995	EPI_ISL_3827971	EPI_ISL_3832367	EPI_ISL_3832470	EPI_ISL_3922231	
EPI_ISL_3802996	EPI_ISL_3827976	EPI_ISL_3832368	EPI_ISL_3832472	EPI_ISL_3922234	
EPI_ISL_3802997	EPI_ISL_3827987	EPI_ISL_3832369	EPI_ISL_3832473	EPI_ISL_3922237	
EPI_ISL_3802998	EPI_ISL_3828020	EPI_ISL_3832370	EPI_ISL_3835245	EPI_ISL_3922242	
EPI_ISL_3802999	EPI_ISL_3832329	EPI_ISL_3832371	EPI_ISL_3835250	EPI_ISL_3922252	
EPI_ISL_3803001	EPI_ISL_3832330	EPI_ISL_3832372	EPI_ISL_3835275	EPI_ISL_3922258	
EPI_ISL_3803002	EPI_ISL_3832331	EPI_ISL_3832373	EPI_ISL_3835279	EPI_ISL_3922260	
